# A case report of refractory multivessel coronary spasm associated with hypereosinophilic syndrome: one cell, one disease?

**DOI:** 10.1093/ehjcr/ytae247

**Published:** 2024-05-15

**Authors:** Shigeo Godo, Hidenobu Takagi, Kohei Komaru, Jun Takahashi, Satoshi Yasuda

**Affiliations:** Department of Cardiovascular Medicine, Tohoku University Graduate School of Medicine, 1-1 Seiryo-machi, Aoba-ku, 980-8574 Sendai, Japan; Department of Diagnostic Radiology, Tohoku University Hospital, Sendai, Japan; Department of Cardiovascular Medicine, Tohoku University Graduate School of Medicine, 1-1 Seiryo-machi, Aoba-ku, 980-8574 Sendai, Japan; Department of Cardiovascular Medicine, Tohoku University Graduate School of Medicine, 1-1 Seiryo-machi, Aoba-ku, 980-8574 Sendai, Japan; Department of Cardiovascular Medicine, Tohoku University Graduate School of Medicine, 1-1 Seiryo-machi, Aoba-ku, 980-8574 Sendai, Japan

**Keywords:** Case report, Coronary artery spasm, Eosinophilia, Hypereosinophilic syndrome, Vasospastic angina

## Abstract

**Background:**

Hypereosinophilic syndrome (HES) is characterized by moderate to severe eosinophilia, exclusion of neoplastic or secondary origins of eosinophilia, and systemic involvement with end-organ damage. Coronary arteries can be affected to cause vasospastic angina (VSA); however, the association of the two diseases is not well recognized.

**Case summary:**

A 55-year-old woman who had a history of multiple allergic disease such as bronchial asthma and chronic sinusitis with nasal polyps was hospitalized due to attacks of chest pain at rest. During a spontaneous attack of chest pain, ECG revealed ST-segment elevation in the inferior leads and emergency coronary angiography showed focal spasms of the right and left anterior descending coronary arteries, both of which were relieved after intracoronary administration of nitroglycerine. She was diagnosed with VSA according to the Japanese Circulation Society guidelines. Despite conventional vasodilator therapies, her resting angina remained refractory. Laboratory findings were notable for moderate eosinophilia. A comprehensive evaluation to uncover the underlying cause of refractory VSA led to the diagnosis of HES, concomitant with eosinophilic pneumonia and eosinophilic chronic rhinosinusitis. Pericoronary inflammation by fat attenuation index (FAI) was increased in the proximal segment of the right coronary artery. Treatment was initiated with oral prednisolone at a starting dose of 30 mg/day. The response to treatment was rapid, with her symptoms disappearing and a regression of eosinophilia observed the following day.

**Discussion:**

Hypereosinophilic syndrome manifests with refractory VSA, and eosinophil-suppressing corticosteroid therapy proves effective in improving both conditions along with reduction of the pericoronary inflammation by FAI.

**Learning points**

- Recognize the potential overlap between refractory vasospastic angina and hypereosinophilic syndrome and acknowledge the role of eosinophilia in the development of refractory vasospastic angina.
- Acknowledge corticosteroids as the fundamental treatment approach in this scenario of ‘one cell, one disease’.
- Highlight the amelioration of pericoronary inflammation surrounding the sites of spasm, as evidenced by the improvement in the fat attenuation index following treatment.

## Introduction

Hypereosinophilic syndrome (HES) is characterized by moderate to severe eosinophilia, exclusion of neoplastic or secondary origins of eosinophilia, and systemic involvement with end-organ damage. Susceptible organs in HES are the heart, lungs, skin, sinuses, nervous system, and gastrointestinal tract. Coronary arteries can be affected to cause coronary spasm and the first case of HES associated with vasospastic angina (VSA) was reported in 1989.^1^ However, the association of the two diseases is not well recognized.

## Summary figure

**Figure:**
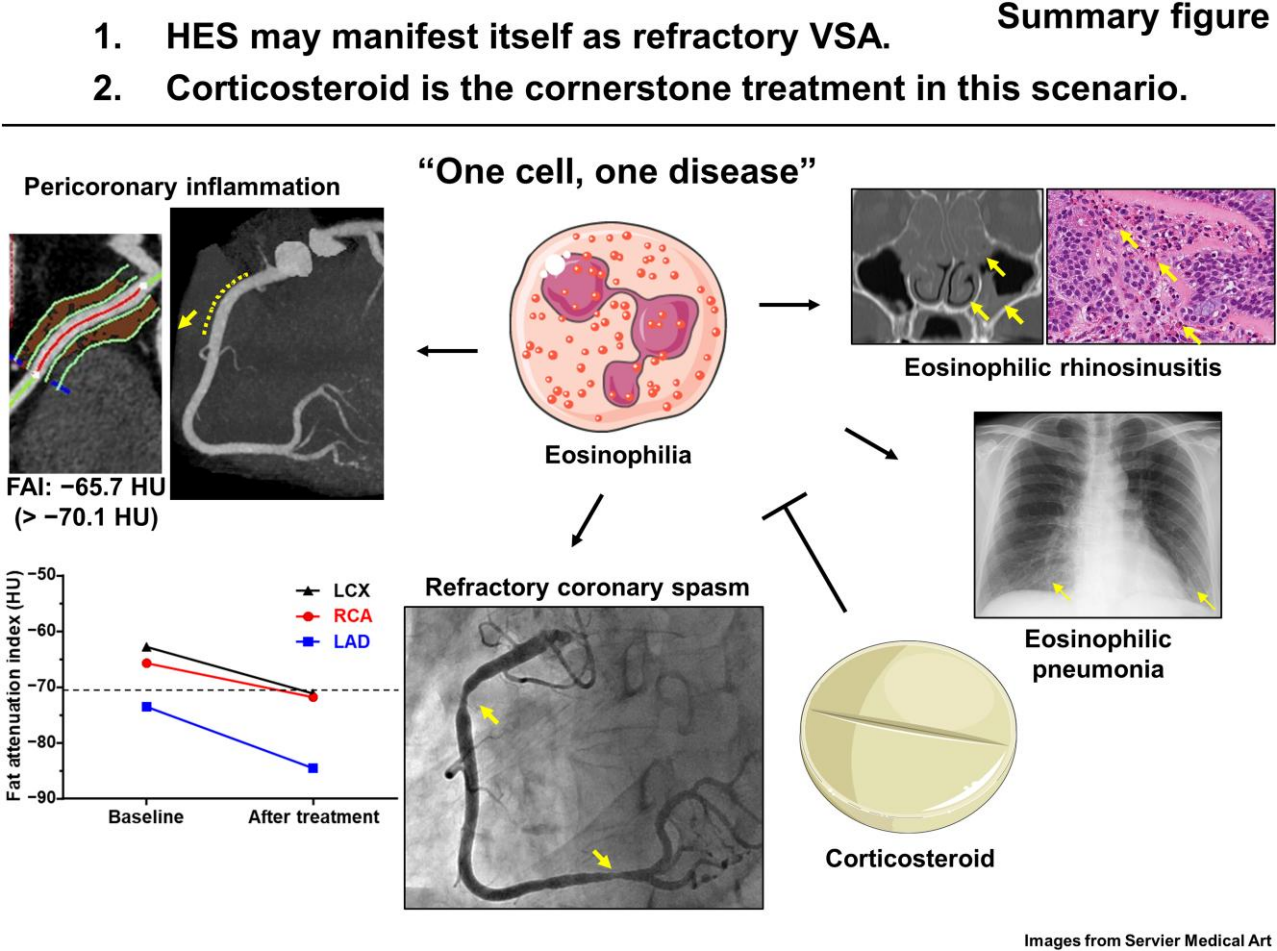


## Case presentation

**Figure 1:**
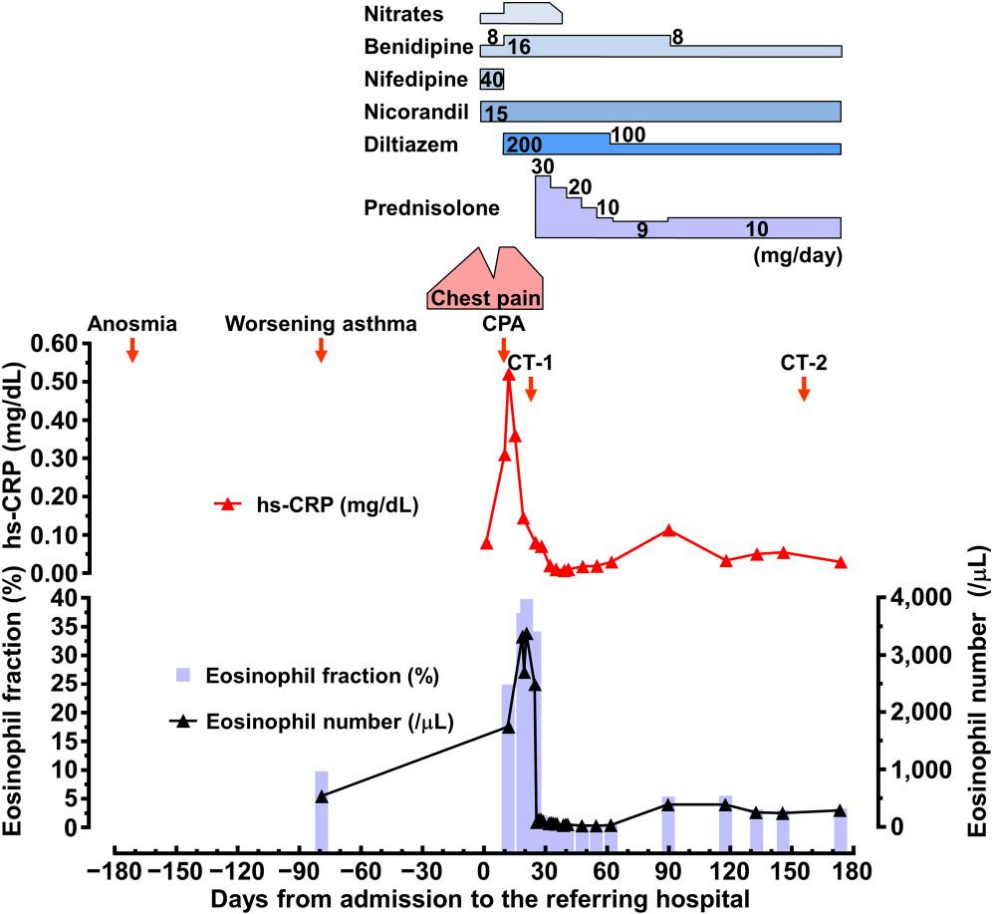
Clinical course. CPA, cardiopulmonary arrest; hs-CRP, high-sensitivity C-reactive protein.

**Figure 2:**
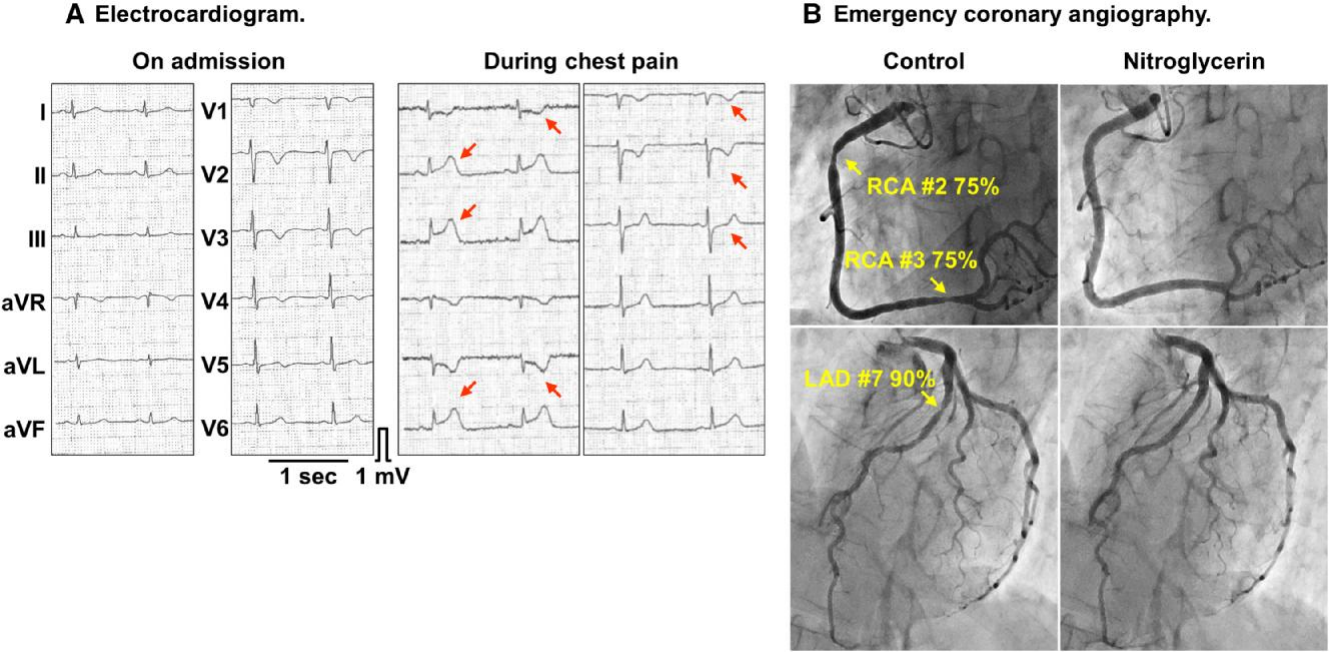
Findings during a spontaneous attack of chest pain. (*A*) Electrocardiogram. (*B*) Emergency coronary angiography.

A 55-year-old woman with a medical history significant for bronchial asthma, allergic rhinitis, chronic sinusitis with nasal polyps, aspirin-sensitive asthma, and urticaria presented with complaints of anosmia, cough, and dyspnoea, accompanied by mild eosinophilia [eosinophil count: 534/μL (9.7%), reference range: 0–520/μL (0–8.5%)] (*Figure 1*). For the decade preceding her presentation, she had been receiving treatment for the mentioned allergic conditions and had remained on a regimen involving multiple anti-allergic medications. Five months later, she began experiencing attacks of chest pain radiating from the left upper extremity to the cervical region, occurring in the morning and evening at rest, with symptoms worsening over time. One month following these episodes, she was hospitalized elsewhere for evaluation of her chest pain. During hospitalization, she had an episode of chest pain at rest, with electrocardiogram (ECG) showing ST-segment elevation in leads II, III, and aVF with reciprocal changes (*Figure 2A*). Emergency coronary angiography revealed focal luminal narrowing of 75–90% in the proximal and distal segments of the right coronary artery (RCA) and the proximal segment of the left anterior descending coronary artery (LAD). These narrowed segments dilated upon intracoronary nitroglycerine administration, consistent with coronary vasospasm (*Figure 2B*). She received a diagnosis of VSA based on the diagnostic criteria outlined in the Japanese Circulation Society 2023 guidelines.^2^ Despite receiving conventional vasodilator therapies, including intravenous nitrates (nitroglycerine 0.5 mg/h), calcium channel blockers (benidipine 16 mg/day, nifedipine 40 mg/day, and diltiazem 200 mg/day), and nicorandil (15 mg/day), her resting angina remained refractory. Subsequently, she experienced cardiac arrest due to pulseless electrical activity during a recurrent episode of chest pain and was successfully resuscitated without neurologic sequelae. Laboratory findings revealed moderate eosinophilia [eosinophil count: 1743/μL (24.9%)]. She was referred to our department for further investigation and management of refractory VSA. Upon admission to our hospital, she was alert and oriented, with a heart rate of 97 beats/min; blood pressure, 99/51 mmHg; body temperature, 37.2°C; normal respiratory rate; and percutaneous oxygen saturation, 97% in ambient air. Her height, weight, and body mass index were 161 cm, 56.5 kg, and 21.8 kg/m^2^, respectively. Physical examination findings were unremarkable.

**Figure 3:**
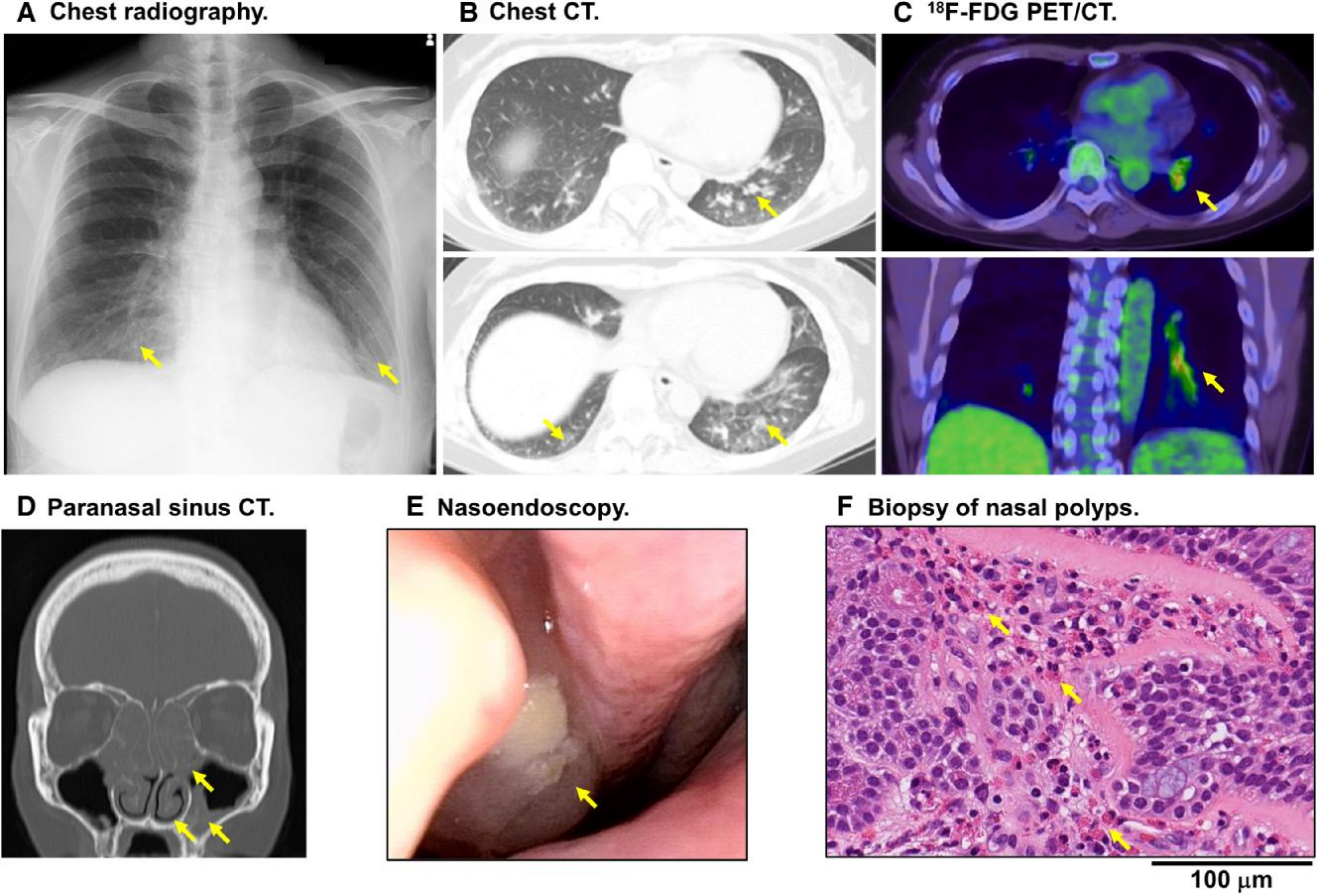
Systemic involvement with hypereosinophilia. (*A*) Chest radiography showing mottled, cord-like opacities within the bilateral lower pulmonary fields. (*B*) Chest CT showing thickened bronchial walls and ground glass opacities in the bilateral lower lobes. (*C*) ^18^F-FDG PET/CT showing thickened bronchial walls in the left lower pulmonary lobe with FDG uptake, accompanied by mild FDG accumulation in the bilateral peripheral lower lobes. (*D*) Paranasal sinus CT showing extensive, high-density opacities within the bilateral nasal cavities, ethmoidal, frontal, sphenoidal, and left maxillary sinuses. (*E*) Nasoendoscopy showing nasal polyps. (*F*) A biopsy of nasal polyps with haematoxylin and eosin staining revealed eosinophilic rhinosinusitis.

**Figure 4:**
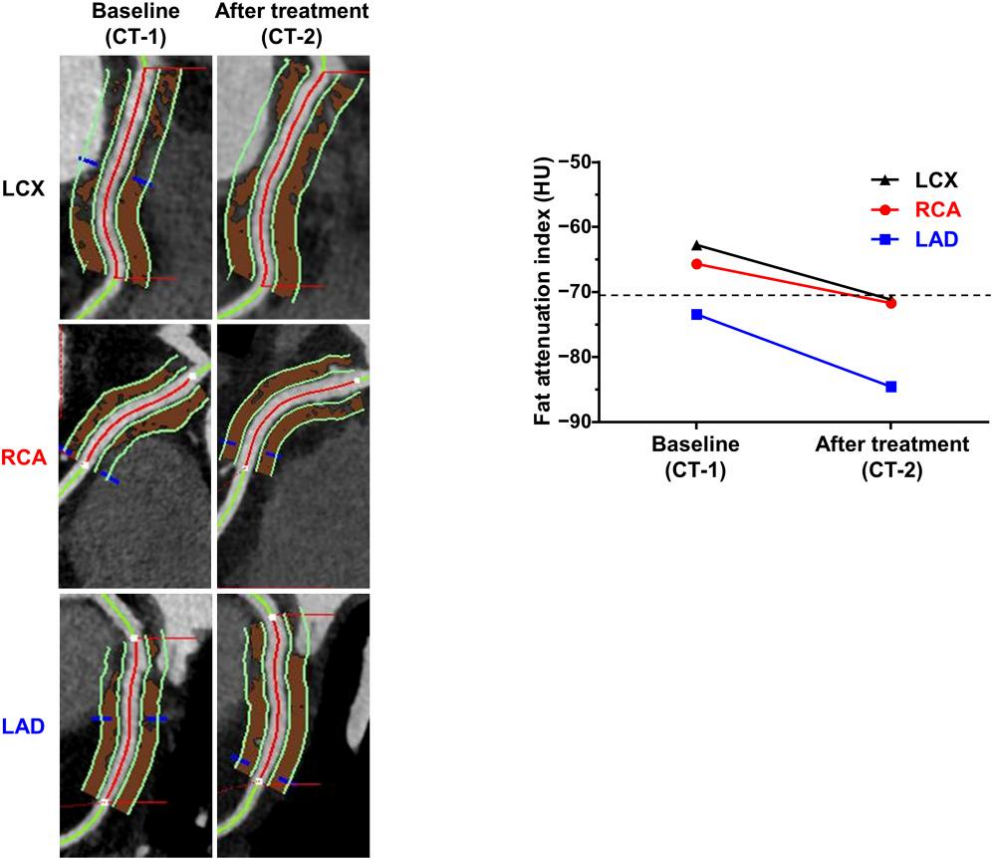
FAI by coronary CT angiography. A higher FAI value is associated with increased coronary inflammation and vice versa. The dotted line indicates a cut-off value (−70.1 HU) for FAI, beyond which cardiac mortality increased in the CRISP CT study.^3^ The FAI values derived from scans performed at 100 kVp were divided by a conversion factor of 1.11485 as described previously.^3^ LCX, left circumflex coronary artery.

Recognizing the lack of response to conventional vasodilator therapies, we embarked on a comprehensive evaluation to elucidate the underlying cause of coronary hyperconstriction. This investigative approach resulted in the diagnosis of HES, concurrent with eosinophilic pneumonia (*Figure 3A*–*C*) and eosinophilic chronic rhinosinusitis (*Figure 3D*–*F*). Laboratory findings revealed significant eosinophilia [eosinophil count: 3320/μL (37.3%)] and elevated serum levels of high-sensitive C-reactive protein (0.145 mg/dL, reference range: ≤ 0.140 mg/dL), while tests for anti-neutrophil cytoplasmic antibodies (ANCA) and the Fip1-like 1-platelet-derived growth factor receptor alpha (FIP1L1-PDGFRA) fusion gene were negative. Secondary causes of eosinophilia, such as drug reactions, vasculitis, collagen disorders, parasitic infections, and Addison’s disease, were ruled out. Echocardiography and spirometry findings were within normal limits. Coronary CT angiography revealed elevated pericoronary fat attenuation index (FAI) levels in the coronary arteries (*Figure 4*).

We initiated immunosuppressive therapy with oral prednisolone at an initial dose of 30 mg/day (∼0.5 mg/kg/day). The response to treatment was swift, with her anginal attacks and anosmia resolving, and a reduction in eosinophilia [80/μL (1.0%)] observed the following day. Subsequently, we tapered the corticosteroid therapy by 5 mg/day every week until reaching a maintenance dose of 10 mg/day. However, when attempting to decrease prednisolone from 10 mg/day to 9 mg/day, the patient experienced a recurrence of anosmia, cough, and chest discomfort, accompanied by a mild increase in eosinophil count [390/µL (5.3%)]. Consequently, the prednisolone dose was reverted to 10 mg/day, leading to symptom improvement. Her clinical course remained uneventful for six months while she was maintained on the dose of prednisolone (10 mg/day). A repeat coronary CT angiography performed five months after treatment initiation demonstrated a significant reduction in pericoronary FAI values (*Figure 4*).

## Discussion

The present case underscores two important clinical implications. Firstly, HES presents with VSA refractory to conventional vasodilator therapies, and corticosteroid therapy aimed at suppressing eosinophil activity proves effective in ameliorating both conditions. Secondly, immunosuppressive treatment with corticosteroids not only alleviates inflammation within the pericoronary adipose tissue, as evaluated by FAI, but also correlates with the resolution of refractory VSA, suggesting an inflammation-mediated mechanism.

The patient presented with refractory VSA alongside allergic diseases and eosinophilia. The differential diagnosis included HES, eosinophilic granulomatosis with polyangiitis (EGPA, formerly known as Churg–Strauss syndrome), and Kounis syndrome. Although some features overlapped with EGPA, such as eosinophilia, systemic symptoms indicative of vasculitis were notably absent, as were specific laboratory markers typically associated with EGPA. Moreover, diagnostic criteria distinguishing HES from EGPA, based on C-reactive protein levels, were not met in this case.^4^ Kounis syndrome, characterized by coronary spasm triggered by allergic or anaphylactoid reactions, typically presents with acute and transient coronary spasm, contrasting with the more chronic nature of vasospasm observed in this patient.

The most valuable lesson gleaned from this case is the efficacy of corticosteroid therapy in managing refractory VSA associated with HES. HES diagnosis hinges on the presence of moderate or severe eosinophilia, exclusion of evident neoplastic or secondary causes of eosinophilia, and systemic involvement, which can affect various organs including the heart, lungs, skin, sinuses, nervous system, gastrointestinal tract, and may even lead to thromboembolic events. Medical management entails two key approaches: firstly, identifying and treating the underlying cause of eosinophilia, and secondly, promptly addressing organ damage associated with HES through corticosteroids and biologic agents to rapidly reduce the eosinophil burden.^5^ In patients resistant or intolerant to corticosteroids, alternative immunosuppressive therapies or biologic agents may be considered.^5^ The first reported case of HES associated with refractory VSA dates back to 1989,^1^ and since then, sporadic case reports have emerged, including cases complicated by EGPA.^6–11^ The clinical characteristics of previously reported cases closely resemble those of our patient, as summarized in *Table 1*.

**Table 1:** Characteristics of previous cases with eosinophilia and vasospastic angina

1. Clinical features
Often occurs in middle age with no gender differences Bronchial asthma and sinusitis are common complications
2. Features of coronary spasm
Multivessel coronary spasm Severity of coronary spasm is linked to that of eosinophilia Refractory to conventional vasodilators but manageable by corticosteroid Provocation tests often require ergonovine instead of acetylcholine Often diagnosed by documenting spontaneous anginal attack
3. Treatment
Early steroid treatment is recommended Prednisolone started at 20–40 mg/day, tapered, maintained at 5–10 mg/day In case of EGPA, more aggressive immunosuppressive therapy may be required Biologics (e.g. anti-immunoglobulin E antibody omalizumab) may be used

Furthermore, the course of this case prompts consideration of the correlation between eosinophilia and VSA. Fat attenuation index is a novel imaging biomarker that can quantitatively evaluate inflammation in pericoronary adipose tissue, and has the characteristic of being a dynamic index compared to conventional indicators such as calcium score.^12,13^ The landmark CRISP CT trial identified a pericoronary FAI of ≥−70.1 HU as a marker associated with heightened risk of cardiac mortality.^3^ Notably, in our case, pericoronary FAI improved beyond this critical threshold following treatment. In addition to previously hypothesized mechanisms of coronary spasm in HES involving eosinophil-derived contracting mediators and neural stimulation from eosinophil infiltration within the coronary adventitia, a mechanism of coronary hyperconstriction mediated by eosinophilic inflammation may be speculated.

## Conclusions

Hypereosinophilic syndrome may present as refractory VSA. Corticosteroid therapy promptly relieved symptoms and normalized eosinophil counts.

## Data Availability

The data underlying this article will be shared on reasonable request to the corresponding author.

## Lead author biography

Dr Shigeo Godo is a Board Certified Member of The Japanese Circulation Society, who obtained his MD and PhD degrees at the Tohoku University School of Medicine in Sendai, Japan. His research interests focus on endothelial function and coronary physiology. He completed research fellowship training in cardiovascular medicine in the Center for Coronary Physiology and Imaging at the Mayo Clinic, Rochester, MN, USA.

## Notes

**Consent:** The authors confirm that written consent for submission and publication of this case report including the images and associated text has been obtained from the patient in line with COPE guidance.

**Funding:** This work was supported in part by the Grant-in-Aid for Scientific Research from the Ministry of Education, Culture, Sports, Science and Technology, Tokyo, Japan (21K16044).
